# The impact of Casemix system on quality of patient care in a Class B hospital in west Sumatera Province, Indonesia

**DOI:** 10.1186/1472-6963-12-S1-O9

**Published:** 2012-11-21

**Authors:** Kamal Kasra, Amrizal Muhammad Nur, Syed Mohamed Aljunid

**Affiliations:** 1 Andalas University of Padang, West Sumatera, Indonesia; 2 United Nations University International Institute for Global Health, Kuala Lumpur, Malaysia

## Background

Health spending in Indonesia is on the increase every year due to a number of factors including change in pattern of diseases towards chronic and non-communicable conditions, priority on curative care rather than preventive services, use of new technology in health services and use of fee-for-service for provider payment. Raise in health care cost can have negative impact on accessibility of health service by the poor communities. In Indonesia, the Ministry of Health has launched a special program to ensure that the poor have access to health care services. One of the most important programs is JAMKESMAS, which is a special health financing scheme for the poor funded by taxation. The implementation of Casemix in Indonesia is based on a special law (Undang-undang No.40) and Minister of Health decree (SK Menkes No. 1663/MENKES/SK/XII/2005). Casemix was implemented as a pilot project involving 14 hospitals in 2005. However in 2008, the use of Casemix was extended to cover all hospitals in Indonesia. Hospitals are reimbursed on locally customized Casemix system called INA-CBG, which is based on UNU-CBG case-mix grouper. In the first phase of Casemix implementation, the system was used to develop hospital tariff for prospective payment with the objective of enhancing service efficiency. However it is widely known that Casemix system is also a powerful tool to enhance quality of care. A number of challenges were identified in the early part of case-mix implementation in Indonesia. These include lack of basic data for Casemix, poor documentation of diagnosis and procedures, and ineligible handwriting of doctors in the medical records. Presently, there is no study carried out in Indonesia to assess the impact of case-mix system implementation on quality of patient care.

## Objective

The aim of the study is to evaluate the impact of Casemix implementation on quality of patient care in one class B hospital in West Sumatera, Indonesia.

## Method

Both qualitative and quantitative methods will be used in this study. The respondents in the study are staff working the hospitals who are directly involved with the JAMKESMAS and case-mix system. This includes doctors, nurses, medical coders and financial officers. Qualitative data will be gathered through in-depth interview with selected respondents. Quantitative data will be collected using self-administrated questionnaires and service observation checklist.

## Conclusion

It is hope that this study will shed lights on the role of Casemix system in improving quality of patient care

